# The Effectiveness of Virtual Reality in Managing Acute Pain and Anxiety for Medical Inpatients: Systematic Review

**DOI:** 10.2196/17980

**Published:** 2020-11-02

**Authors:** Vinayak Smith, Ritesh Rikain Warty, Joel Arun Sursas, Olivia Payne, Amrish Nair, Sathya Krishnan, Fabricio da Silva Costa, Euan Morrison Wallace, Beverley Vollenhoven

**Affiliations:** 1 Department of Obstetrics and Gynaecology Monash University Clayton Australia; 2 Biorithm Pte Ltd Singapore Singapore; 3 Department of Paediatrics West Gippsland Hospital Warragul Australia; 4 Department of Gynecology and Obstetrics Ribeirão Preto Medical School Sao Paulo Brazil

**Keywords:** virtual reality, VR, pain management, anxiety, procedural pain, wound management, acute pain, analgesia, pain

## Abstract

**Background:**

Virtual reality is increasingly being utilized by clinicians to facilitate analgesia and anxiolysis within an inpatient setting. There is however, a lack of a clinically relevant review to guide its use for this purpose.

**Objective:**

To systematically review the current evidence for the efficacy of virtual reality as an analgesic in the management of acute pain and anxiolysis in an inpatient setting.

**Methods:**

A comprehensive search was conducted up to and including January 2019 on PubMed, Ovid Medline, EMBASE, and Cochrane Database of Systematic reviews according to PRISMA (Preferred Reporting Items for Systematic Reviews and Meta-Analyses) guidelines. Search terms included virtual reality, vr, and pain. Primary articles with a focus on acute pain in the clinical setting were considered for the review. Primary outcome measures included degree of analgesia afforded by virtual reality therapy, degree of anxiolysis afforded by virtual reality therapy, effect of virtual reality on physiological parameters, side effects precipitated by virtual reality, virtual reality content type, and type of equipment utilized.

**Results:**

Eighteen studies were deemed eligible for inclusion in this systematic review; 67% (12/18) of studies demonstrated significant reductions in pain with the utilization of virtual reality; 44% (8/18) of studies assessed the effects of virtual reality on procedural anxiety, with 50% (4/8) of these demonstrating significant reductions; 28% (5/18) of studies screened for side effects with incidence rates of 0.5% to 8%; 39% (7/18) of studies evaluated the effects of virtual reality on autonomic arousal as a biomarker of pain, with 29% (2/7) demonstrating significant changes; 100% (18/18) of studies utilized a head mounted display to deliver virtual reality therapy, with 50% being in active form (participants interacting with the environment) and 50% being in passive form (participants observing the content only).

**Conclusions:**

Available evidence suggests that virtual reality therapy can be applied to facilitate analgesia for acute pain in a variety of inpatient settings. Its effects, however, are likely to vary by patient population and indication. This highlights the need for individualized pilot testing of virtual reality therapy’s effects for each specific clinical use case rather than generalizing its use for the broad indication of facilitating analgesia. In addition, virtual reality therapy has the added potential of concurrently providing procedural anxiolysis, thereby improving patient experience and cooperation, while being associated with a low incidence of side effects (nausea, vomiting, eye strain, and dizziness). Furthermore, findings indicated a head mounted display should be utilized to deliver virtual reality therapy in a clinical setting with a slight preference for active over passive virtual reality for analgesia. There, however, appears to be insufficient evidence to substantiate the effect of virtual reality on autonomic arousal, and this should be considered at best to be for investigational uses, at present.

## Introduction

### Background

The International Association for the Study of Pain defines pain as an “unpleasant sensory and emotional experience associated with actual or potential tissue damage [1].” As a disease, pain carries a huge global burden with a prevalence of 20% and incidence of 10%. It negatively affects one’s psychological and social functioning, thereby impinging on quality of life too. Its tangible costs cannot be understated as well, with research demonstrating a loss in productivity owing to absenteeism and diminished job performance as a result [2-7].

Acute pain is a commonly encountered clinical entity in up to 84% of patients presenting to medical services [8]. Acute pain is sudden in its onset and is typically expected to last for a short time (≤6 weeks). Usually, it can be attributed to a specific event or illness, but at times, it may be iatrogenic [9].

Either pharmacologic (analgesics) and nonpharmacologic (interventions) can be used for addressing acute pain. Within analgesics, opioids are often prescribed [10]. This strategy, although clinically effective, has several disadvantages. Opioids are notorious for their deleterious side effects, including tolerance, dependence, and hyperalgesia [11]. Additionally, some analgesics also require invasive procedures to be administered, such as with intrathecal infusions, which carry their own set of clinical risks and side effects. Similarly, concerns have been raised toward nonpharmacologic approaches (ie, transcutaneous electrical nerve stimulation, hot or cold compress), with regard to their efficacy and appropriateness in the setting of acute pain [10,11].

In light of these findings, as well as the recent recommendations from the American Pain Society and the American Society of Anaesthesiologists, there remains an urgent need to characterize and identify alternative modalities for acute pain relief. In particular, there is a need for these therapies to be clinical efficacious, be minimally invasive, and potentiate low levels or negligible side effects [12]

Virtual reality is a burgeoning technology which is in its infancy of uptake for clinical utilization. As a technology, virtual reality allows for users to be immersed in a virtual environment through multisensorial stimulation [13,14].

Over the last decade, increasing attention and research has been directed toward assessing the utility of virtual reality in managing acute pain. While there is presently no clear explanation of virtual reality’s mechanism of pain relief available, several theories which span the realms of biology and psychology exist to elucidate its efficacy [15,16]. To date, it is also worthy of mention that virtual reality therapy has been successfully used as an analgesic in several acute clinical contexts, ranging from pediatric phlebotomy to dressing changes for burns and postcardiac surgery [17-19].

### Objectives

The main motivation for our group in undertaking this systematic review was to provide a comprehensive literature review to inform the clinical utilization and testing of virtual reality therapy.

Primarily, this encompassed understanding the applicability of virtual reality in facilitating analgesia during acute pain for inpatient populations. This also included an understanding of virtual reality therapy headsets, content being used for the indication, and the effects of virtual reality on anxiolysis, since it has been implicated in facilitating this effect, which in turn, modulates the patient’s perception of pain [20,21]. Furthermore, we aimed to delineate the effect of virtual reality on physiological parameters (autonomic arousal); literature has suggested that these are biomarkers of pain, and thus, are theoretically correlated with pain responses [22]. Last, given the impact that it would have on patient safety and clinical uptake, we wanted to understand virtual reality therapy’s side effect profile.

Prior to undertaking this review, there was a gap in the literature on virtual reality therapy, in the setting of facilitating inpatient acute analgesia, which was of practical relevance to the clinician. This, in our opinion, appeared to be a barrier of clinical uptake which we aimed to address through this initiative by providing a holistic overview.

## Methods

### Data Sources

The following review was conducted in line with PRISMA (Preferred Reporting Items for Systematic Reviews and Meta-Analyses) guidelines. The search was undertaken on PubMed, Ovid Medline, EMBASE, and the Cochrane Database of Systematic Reviews up to and including May 2018 by VS, JS, AN, and SK and repeated up to and including January 2019 by VS, RRW, OP, and JS. The search was carried out without any limit of the years, and articles were restricted to those in English. The databases were searched independently by the aforementioned authors. Once shortlisted, full texts were ordered and read. The bibliographies of articles selected for the review were also screened for suitable additional articles to be included in this review. Inclusion in the review was selected by consensus between the screening authors.

Inclusion criteria were primary studies utilizing virtual reality in the management of acute pain in a clinical setting. Interventions were considered to be virtual reality only if they employed an audio or visual multimedia environment with which the patients could view or interact (ie, games and videos). Acute pain was defined as pain that was less than 6 weeks in duration and associated with an acute condition or medical procedure. The context was selected to make the findings relevant to the inpatient treatment of patients for clinicians. Studies with both adult and pediatric populations were suitable for inclusion in the review.

Articles that were reviews, case series, or case reports were excluded from this review. Experimental studies of a nonclinical nature (eg, pain induced via cold-pressor test) were excluded in a bid to focus on clinically relevant pain reduction which could be easily extrapolated to clinical practice.

### Search Strategy

Search terms used across all databases were (*virtual reality* OR *vr*) AND *pain*. Studies were then filtered manually as per the inclusion criteria for acute pain associated with acute conditions or procedures.

### Data Collection Process

Data were extracted manually for analysis by VS, SK, and JA in tabular form. Due to the heterogeneity of the studied populations, variations in technologies utilized, and heterogeneity in the endpoints of the studies; pooling of data for meta-analysis was not considered appropriate. In addition, meta-analysis of data from rapidly evolving medical technologies of various generations was deemed inappropriate [23]. This is due to the lack of similarity between technologies, the impact of incremental innovation between generations of the same technology and the presence of operator dependence (from a clinician and patient perspective) on its performance [23]. As such, a narrative approach was followed for this review.

### Data Items

Data items of interest for the studies included: year of study, study design, sample size, clinical setting, population, nature of the intervention, control or comparison, virtual reality content type, main outcomes measures, outcome measurement tools, and technical specifications of virtual reality devices employed. Technical specifications of virtual reality devices included type of head mounted display, display utilized, weight of device, field of view, computer, video card, controller, virtual reality content type, and virtual reality content used.

### Assessment of Bias

The risk of bias was assessed by VS and RRW using the modified Downs and Black List [24] and scored on a scale of 1-10 as illustrated in Table 1.

**Table 1:** Summary of bias assessment results using modified Downs and Black checklist.

Study	Score (out of 10)
Chad et al [25]	9
Chan et al [26]	6
Chau et al [27]	8
Frey et al [28]	9
Gerceker et al [29]	10
Gershon et al [30]	8
Glennon et al [31]	9
Gold et al [32]	9
Hoffman et al [18]	8
McSherry et al [33]	7
Mosso-Vasquez et al [17]	8
Mosso-Vasquez et al [34]	7
Nilsson et al [19]	9
Piskorz et al [35]	8
Shoorab et al [36]	9
Tashjian et al [37]	9
Walker et al [38]	8
Yun Hua et al [39]	9

### Summary Measures and Synthesis of Results

The primary measures of interest were degree of analgesia afforded by virtual reality therapy, degree of anxiolysis afforded by virtual reality therapy, effect of virtual reality on physiological parameters, side effects precipitated by virtual reality, measures of pain assessment, virtual reality content type, and types of equipment utilized.

### Patient and Public Involvement

No patients were involved in the design, recruitment, or conduct of the study. There was no intention a priori that the results of this review would be disseminated to patients included in the trials of the review.

## Results

### Study Characteristics

#### General Description of Studies

**Figure 1:**
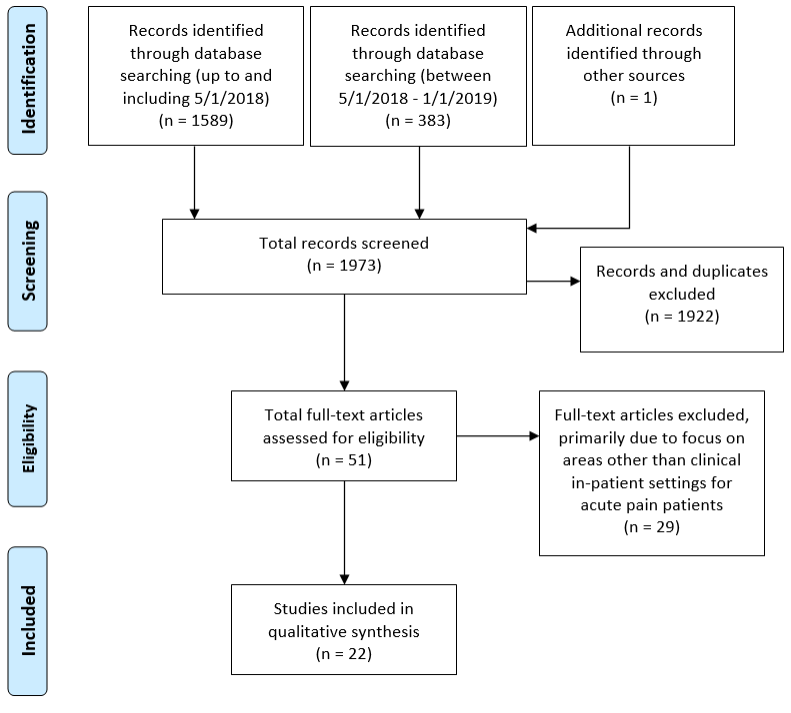
PRISMA flow diagram.

A total of 18 studies were deemed suitable for inclusion in this review [17-19,25-39]. The article selection process is outlined in Figure 1.

The descriptive data collated from the eligible studies are reported in Table S1 (Multimedia Appendix 1). All 18 studies were conducted and published between 2004 and 2018: 50% (9/18) of studies were specifically focused on the pediatric population; 72% (13/18) of studies compared virtual reality against standard analgesia as the comparator/control group [18,19,26,29-36,38,39]; 28% (5/18) of studies compared virtual reality to no analgesia [17,25,27,28,37]. Overall, only 1 study of 18 (6%) received a bias assessment score less than 7 out of 10.

#### Indications for Virtual Reality Utilization

Of the 18, 22% (4 studies) measured pain reduction in patients undergoing dressing changes or hydrotherapy for burns or wounds [18,26,33,39]; 39% (7 studies) looked at patients undergoing venipuncture, port access, or injections [19,25,27,29,30,32,35]; 39% (7 studies) measured pain outcomes in patients undergoing various invasive procedures (5 studies) [17,31,34,36,38] or other miscellaneous acute conditions (2 studies) [28,37].

#### Technical Specifications of Virtual Reality Systems Utilized

Table S2 (Multimedia Appendix 2) summarizes data on the technical specifications of devices.

All studies (18/18, 100%) utilized head mounted displays which allowed for monitoring of head orientation. Half (9/18, 50%) used active virtual reality content in the form of games [18,19,26,30,32,33,35,38,39] as the primary content, and the remaining 50% of the studies (9/18) employed passive virtual reality content in the form of virtual environments and movies. One-third (6/18, 33%) applied a virtual environment [17,25,28,31,34,37], and 17% (3/18) displayed a cartoon or movie [27,29,36].

### Summary of Measures and Synthesis of Results

#### Effect on Analgesia

Overall, 67% (12/18) of studies in this review demonstrated a statistically significant reduction in pain during virtual reality utilization; 83% (10/12) of these demonstrated a statistically significant reduction in pain using a between-group design [18,26,28-30,32,35-37,39]. Within these 10 studies, only 1 received a score less than 7 on the bias assessment checklist. The remaining 17% (2/12) of studies demonstrated statistically significant reductions in perceived pain utilizing a within-group design [25,34]. Across studies, the clinical endpoint of pain was measured through a variety of pain measurement tools. These results and tools are described in Table 2.

**Table 2:** Summary of studies for utility of virtual reality in acute pain.

Study	Intervention	Standard care; control	Measurement tools	Results
Chad et al [25]	VR^a^	N/A^b^	FACES^c^ (scale of 0-5)	Descriptive evidence of significantly reduced pain as reported by parents: 83% reduction (*P=*.02) in pain noted pre-VR (mean 3.34, SD 1.30) and post-VR (mean 0.76, SD 1.06). Insignificant reduction in pain reported by children: 77% reduction in pain noted with VR use (mean difference –2.5; *P=*.52). No information on pain score from children and variance.
Chan et al [26]	VR	Routine analgesia	FACES (scale of 0-100)	Significant differences (*P<*.05) of pain intensity found between VR group and non-VR group with ANOVA^d^: pain during procedure (VR: mean 38.13, SD 12.02; control: mean 53.75, SD 11.80) and pain after procedure (VR: mean 8.75, SD 2.95; control: mean 18.75, SD 9.53).
Chau et al [27]	VR	N/A	FLACC^e^ (scale of 0-10)	FLACC scores with VR use: median 2.5 (1-5.5); range (1-8). 64.3% (9/14) of caregivers felt that the VR experience was positive and wanted to use it again. No between group differences explored for the following study. Descriptive evidence of reduced pain as reported by parents.
Frey et al [28]	VR	Unmedicated labor	VNS^f^ (scale of 0-10)	Worst pain intensity was significantly lower in VR vs control (–1.5 (95% CI, –0.8 to –2.2). Significant differences in difference of pain intensity found between VR group and non-VR group with ANOVA.
Gerceker et al [29]	VR	External cold and vibration (buzzy); analgesia alone	Wong Baker FACES (scale of 0-10)	There was a statistically significant difference observed in pain scores between the VR group and control as reported by the patients (VR: mean 1.5, SD 0.2; control: mean 5.1, SD 0.4; *P<*.01) and parents (VR: mean 1.5, SD 0.2; control: mean 4.7, SD 0.4; *P<*.01).
Gershon et al [30]	VR with topical anesthetic	Video game with topical anesthetic; topical anesthetic	VAS^g^ (scale of 0-100); CHEO^h^ pain scale	There was a significant reduction in pain in the VR and non-VR distraction group as observed by nurses (*P<*.05) based on MANOVA^i^. No summary measures were provided in the paper. From the CHEO pain scale, the VR group had significantly fewer behavioral markers in comparison to those of the controls for pain (*P<*.05). No summary measures were provided in the paper. No summary statistics on pain score from children and variance.
Glennon et al [31]	VR	Analgesia alone	NPS^j^ (scale of 0-10)	Participants in the VR group (mean 3.9, SD 2.3)did not experience a statistically significant decrease (*P<*.05) in pain and anxiety in comparison to controls (mean 4, SD 2.7). Not powered adequately. Recruited individuals with prior exposure to bone marrow biopsy which may have skewed the reporting of pain.
Gold et al [32]	VR and analgesia	Analgesia alone	VAS (scale of 0-10); CAS^k^; FACES	Significantly less pain (*P<*.05) was reported by the VR group (mean 1.31, SD 1.59) compared to that reported by the control group (mean 1.93, SD 2.22).
Hoffman et al [18]	VR and analgesia	Analgesia alone	GRS^l^	Statistically significant reduction (*P<*.01) in pain observed in VR (mean 7.6, SD 1.9) vs control (mean 5.1, SD 2.6).
McSherry et al [33]	VR and analgesia	Analgesia alone	VNS; opioid administration	Total opioid administration during VR therapy was significantly less than that when no VR was used (VR: 91.7 SD 10.1; no VR: 103.1 SD 16.1 μg/kg; *P<*.05). Overall, 39% less opioids were used with VR therapy. Pain scores (post procedure – pre procedure) were not significantly (*P*>.05) reduced in VR group (mean difference –1.2, SD 2.9) vs control (mean difference –0.3, SD 1.7).
Mosso-Vasquez et al [17]	VR and analgesia	N/A	VNS	88% of patients reported a reduction in pain after VR therapy. Mean change in the Likert pain scale was 3.75. No descriptive statistics were provided. Change in pain scores (post procedure – pre procedure) was substantially correlated with change in respiratory rate (*R*^2^=0.26). It was, however, minimally correlated with heart rate (*R*^2^=0.05), mean arterial pressure (*R*^2^=0.09), and SpO_2_^m^ (*R*^2^=0.00).
Mosso-Vasquez et al [34]	VR and analgesia	Mobile VR and analgesia	VAS	Overall, both head mounted display (presurgery: 6.06; postsurgery: 1.73) and mobile groups (presurgery 3.78; postsurgery 0.64) showed significant reductions (*P<*.01) in pain with VR. Head mounted display VR group experienced a significantly greater pain reduction from intra to postoperative states in comparison to the mobile VR group (–1.5 vs –0.07; *P*=.02).
Nilsson et al [19]	VR and analgesia	Analgesia alone	CAS; FAS^n^; FLACC	No significant difference in CAS, FAS, and FLACC scores between VR and non-VR groups (*P*>.05). No descriptive statistics were provided.
Piskorz et al [35]	VR and analgesia	Analgesia alone	VAS	The VR group (mean 15.16, SD 20.51) reported significantly lower (*P<*.02) pain intensity compared to that of the control group (37.05 SD 30.66). Pain intensity was 59% lower in the VR group than in the control with a large effect size (Cohen *d*= 0.86).
Shoorab et al [36]	VR and analgesia	Analgesia alone	VNS	Statistically significant reduction in the pain scores were observed during episiotomy repair in the VR group using ANOVA (VR effect: *f*=88.6, *df*=1, *P<*.01). VR group had lower pain scores during several phases of the procedure in comparison to those of the non-VR group (*P<*.0001): during the repair of the hymen (VR: mean 9.0, SD 12.6; non-VR: mean 23.6, SD 19.8), skin (VR: mean 16.7, SD 16.5; non-VR: mean 39.3, SD 22.5), and after the repair (VR: mean 6.0, SD 12.8; non-VR: mean 25.2, SD 14).
Tashjian et al [37]	VR	Nature video	VNS	Pain reduction in the VR group (preintervention: mean 5.4, SD 2.6; postintervention: mean 4.1, SD 2.7) was greater (percentage reduction: 24% vs 12.2%, *P<*.01) than that in the control group within subjects (preintervention: mean 5.4, SD 2.6; postintervention: mean 4.8 SD 2.7). Higher number of responders in VR in comparison to control (≥0.5 SD drop in pain) (65% vs 40%, *P<*.01).
Walker et al [38]	VR and analgesia	Analgesia alone	VAS	No significant difference in pain scores (*P*>.05) between VR group and control—average pain (VR: 44 mm; control: 43 mm) and worst pain (VR: 66 mm; control: 59 mm)—during the procedure.
Yun Hua et al [39]	VR and analgesia	Analgesia alone	FACES;VAS; FLACC	Significantly less pain reported in the VR group compared to the control group before, during and after the dressing change (*P<*.05). Also, significantly lower scores during dressing change in VR vs control (*P<*.05): FACES—VR: mean 2.42, SD 1.85; control: mean 4.19, SD 2.12) VAS—VR: mean 4.35, SD 2.64; control: mean 6.25, SD 2.84) FLACC—VR: mean 4.18, SD 2.97; control: 7.36, SD 3.47).

^a^VR: virtual reality.

^b^N/A: Not applicable.

^c^FACES: facial analysis scale (such as the Wong-Baker Faces Scale).

^d^ANOVA: analysis of variance.

^e^FLACC: Face, Legs, Activity, Cry, Consolability.

^f^VNS: verbal numerical scale.

^g^VAS: visual analog scale.

^h^CHEO: Children’s Eastern Ontario Hospital Pain Scale.

^i^MANOVA: multivariate analysis of variance.

^j^NPS: numerical pain scale

^k^CAS: color analog scale

^l^GRS: graphical representation scale.

^m^SpO_2_: oxygen saturation.

^n^FAS: facial affective scale

#### Effect on Anxiety

Anxiety or stress was measured as a primary outcome in 44% of studies (8/18) [25,28,30-33,35,38]. Within these, 50% (4/8) demonstrated a statistically significant reduction in anxiety; 37.5% (3/8) demonstrated a statistically significant reduction in anxiety utilizing a between-group design [28,32,35]; and 12.5% (1/8) showed a statistically significant reduction in anxiety by means of a within-group design [25]. These results and the tools utilized to measure them are detailed in Table 3.

**Table 3:** Summary measures of studies in review which measured forms of anxiety.

Study	Measurement tools	Results
Chad et al [25]	McMurtry children’s fear scale	Significant reduction in fear detected by parent due to VR^a^ (mean 2.18; *P*=.05). Insignificant reduction in fear reported by child due to VR (mean 2.57; *P*=.43).
Frey et al [28]	VNS^b^ (scale of 0-10)	Anxiety was significantly decreased –1.5 (95% CI –0.8 to –2.3) in the VR condition compared to that in the control condition. Significant difference in anxiety found between VR group and non-VR group using ANOVA^c^.
Gershon et al [30]	VAS^d^ (scale of 0-100); CHEO^e^ Pain Scale	From the CHEO pain scale measure, the VR group had significantly fewer behavioral markers in comparison to controls for anxiety (*P<*.05). No summary measures were provided in paper.
Glennon et al [31]	5-point Likert scale for anxiety	Participants in the VR group did not experience a statistically significant decrease in anxiety in comparison to that in controls (*P*>.05).
Gold et al [32]	VAS (scale of 0-10); FAS^f^	Significantly less anxiety (*P<*.05)was reported and observed in the VR group (mean 1.90, SD 2.2) compared to that in the control group (mean 2.48, SD 2.07).
McSherry et al [33]	VNS	Anxiety scores were not significantly reduced (*P*>.05) in VR group (mean difference –1.3, SD 4.4) vs control (mean difference –0.4, SD 2.7).
Piskorz et al [35]	VAS	The VR group (mean 11.16, SD 18.58) reported significantly lower stress levels (*P<*.01) compared to those in the control group (mean 41.89, SD 40.89). Stress levels were 73.4% lower in VR group against control with a large effect size (Cohen *d*= 0.993).
Walker et al [38]	VAS	No significant difference between intraprocedural anxiety levels. No descriptive statistics were provided.

^a^VR: virtual reality.

^b^VNS: verbal numerical scale.

^c^ANOVA: analysis of variance.

^d^VAS: visual analog scale.

^e^CHEO: Children’s Eastern Ontario Hospital.

^f^FAS: facial affective scale.

#### Effect on Physiological Parameters

The effect of virtual reality on physiological indicators of pain was investigated in 39% of studies (7/18) [17,19,30,34,37-39]; however, the parameters investigated varied between studies, encompassing measures such as heart rate, respiratory rate, oxygen saturation, galvanic skin response, blood pressure, and mean arterial pressure.

Of these, 29% of studies (2/7) demonstrated a significantly reduced heart rate in children or adolescents undergoing virtual reality therapy compared with that of the control group [30,39]. A summary of the results is presented in Table 4.

**Table 4:** Summary of physiological measures and side effects of studies in this review.

Study	Outcome measures	Measurement tools	Results
Frey et al [28]	Nausea; side effects	Questionnaire	No adverse effects reported. No significant differences in occurrences of nausea between VR^a^ and control.
Gershon et al [30]	Physiology	Heart rate	Significant reduction in physiological parameters (heart rate) observed in VR group vs non-VR group vs that of the control during procedure (96.3 vs 103.8 vs 110.3 beats per minute, *P<*.05).
Gold et al [32]	Side effects	Likert scale (scale 1-6)	5.2% (n=4) of patients reported nausea, and 8% reported simulator sickness.
Hoffman et al [18]	Nausea	GRS^b^	Nausea ratings were negligible.
Mosso-Vasquez et al [17]	Side effects; physiology	Questionnaire; heart rate; mean arterial pressure; respiration rate; SpO_2_^c^	Change in pain scores (postprocedure – preprocedure) was minimally correlated with heart rate (*R*^2^=0.05), mean arterial pressure (*R*^2^=0.09), and SpO_2_ (*R*^2^=0.00). 37.3% (25/67) of patients had reduced heart rate after VR therapy. 52.2% (35/67) of patients had reduced mean arterial pressure after VR therapy. 64% (14/22) of patients had reduced respiratory rate after VR therapy. None of these data were tested for statistical significance. 4.5% experienced side effects.
Mosso-Vasquez et al [34]	Physiology	Blood pressure	No significant change in systolic or diastolic blood pressure with VR use.
Nilsson et al [19]	Physiology	Heart rate	No statistically significant difference in heart rate between VR and control group.
Tashjian et al [37]	Physiology; side effects	Questionnaire; blood pressure; heart rate	No adverse side effects reported. No statistically significant differences between systolic blood pressure, diastolic blood pressure, and heart rate pre- and post-VR (*P*>.05).
Walker et al [38]	Physiology; side effects	Questionnaire; heart rate; respiration rate; blood pressure; galvanic skin response	No significant difference between vital signs or galvanic skin response detected. No descriptive data provided. No side effects reported.
Yun Hua et al [39]	Physiology	Heart rate; SpO_2_	Significantly lower heart rate was observed in the VR group compared to the control group (98.88 SD 11.57 vs 106.2 SD 11.45 beats per minute, *P<*.05). No difference in SpO_2_.

^a^VR: virtual reality.

^b^GRS: graphic rating scale.

^c^SpO_2_: oxygen saturation.

#### Side Effects

Of the eligible studies, 33% (6/18) assessed patients for side effects incurred from virtual reality therapy [17,18,28,32,37,38]. The main side effects that were screened were include nausea, vomiting, and vertigo. Overall, the prevalence of side-effects was low and ranged from 0% to 8%. This data is summarized in Table 4.

## Discussion

### Effect on Analgesia

The findings of this review illustrated that there was a significant reduction of pain related to virtual reality therapy utilization in 67% of the studies (12/18). Although acknowledging limitations in interpreting these findings (see limitations below), this evidence is corroborated by the findings of other high-quality studies [40-42], supporting the use of virtual reality therapy as a nonpharmacologic adjunct in facilitating analgesia within a clinical context. An effort was also made to critically appraise the studies which failed to demonstrate any significant differences in pain (Multimedia Appendix 3). The intention here was to examine the studies for factors which may have contributed toward null findings and were considered to be limitations of the study by the authors themselves.

To the practicing clinician, these findings are of relevance as they suggest that virtual reality therapy can be considered as an inpatient adjunct for acute pain, particularly in the context of facilitating procedural analgesia (12/18). However, it is likely that this performance will vary by indication and the patient population to which it is being applied. This variability in performance, therefore, should prompt consideration toward pilot testing, as an initial step, for any specific clinical use in order to establish its appropriateness as a therapeutic modality.

To further elaborate, although the exact mechanisms behind how virtual reality facilitates analgesia are still unknown; there are several plausible theories which may explain its therapeutic effect. One school of thought suggests that virtual reality therapy enacts changes on a neurobiological level, and thereby, facilitates analgesia in a manner similar to a drug. Functional magnetic resonance imaging has been utilized to demonstrate this in experimental models [43]. During episodes of pain stimulus, areas of the neuroanatomic pain matrix (insula, anterior cingulate cortex, thalamus, primary and secondary somatosensory cortices) demonstrated increased levels of activity. When virtual reality therapy is administered to patients during these episodes, a reduction greater than 50% is observed in the activity of the pain matrix, which corresponds with a fall in patient-reported pain ratings [43]. Similarly, experimental models have also demonstrated that this analgesic effect of virtual reality therapy can be controlled in a dose-dependent fashion [44-46].

Alternatively, it has also been theorized that virtual reality enacts its functions on a psychological level through the distraction it provides. The Gate Control Theory [16] proposes that the amount of attention given to a painful stimulus affects the person’s interpretation of it. In line with this, the Multiple Resource Theory [15] also suggests that humans have a finite capacity to provide attention toward and process pain. As such, it is plausible that by rerouting or drawing these mental faculties away from the noxious stimulus, through a mechanism such as virtual reality therapy, that this would successfully attenuate the perception of pain [42,47,48].

### Effect on Anxiety

Within the brain, the limbic system and amygdala are implicated in mediating anxiety, and this is often experienced by patients prior to a medical procedure [49,50]. While the ability to be anxious is essential for survival, increased levels of anxiety in a clinical environment can lead to worsening perceptions of pain, decreased thresholds for pain, and less cooperative patients [20,21]. As such, a rationale does exist for controlling anxiety in the context of facilitating analgesia for patients.

The findings of the review with regard to anxiolysis were equivocal, with 50% (9/18) of the studies demonstrating a significant anxiolytic effect. A recent systematic review [51] demonstrated significantly reduced anxiety scores in individuals undergoing virtual reality therapy for treatment of anxiety disorders in comparison to those of controls. Similarly, there is also some suggestion that virtual reality therapy generates positive emotions and improvements in mood which dampen preprocedural patient anxiety. Also, it is supposed that similar to its analgesic effects, anxiolytic properties occur as a result of the abovementioned psychological alterations [52-54].

There appears to be merit in further evaluating virtual reality therapy for its anxiolytic effect. Particularly, as the potential benefits of anxiolysis extend beyond the mitigation of procedural pain to include an improved patient experience [55].

### Effect on Physiological Parameters

Several studies have attempted to utilize changes in physiological markers or autonomic arousal as surrogate marker of analgesic effect [56]. From a biological perspective, this is not surprising since acute pain activates the sympathoadrenal fight or flight response, which in turn produces autonomic arousal effects (ie, increased respiratory rate, heart rate, blood pressure, skin sweating—galvanic skin response) [22,56-58].

In this review, 39% of studies (7/18) explored the relationship between virtual reality therapy–facilitated analgesia and its effect on a variety of physiological parameters. Although our findings somewhat suggested that heart rate correlated with pain scores [30,39], it was not possible to consistently ascribe utility toward using autonomic arousal as a surrogate marker for analgesic effect. There were several reasons for this.

First, parameters utilized and investigated across studies appeared to be heterogeneous and inconsistent, making it difficult to draw firm conclusions. Next, there was also evidence to suggest that not all physiological markers respond similarly to pain stimulus and subsequent analgesia [17,19,30,34,37-39]. Additionally, it is also known that arousal induced by pain is not static as the participant may be able to influence it either consciously or subconsciously by utilizing their own coping strategies (ie, heightened respiratory rate can be consciously altered by slowing down one’s breathing) [56,58,59]. This, therefore, will arguably impact the ability of physiological markers to be precise and consistent markers of pain. However, it is worth mentioning that the literature seems to suggest that both respiratory rate and galvanic skin response appear to be consistent markers of pain response, whereas cardiovascular changes appear to be less useful [56,58].

Considering these findings, it is safe to say that there is no firm evidence to suggest that virtual reality therapy can either affect autonomic arousal or demonstrate its analgesic properties through modulation of these parameters. Testing these parameters in a uniform and consistent manner, at least within a research context, is merited.

### Side Effects

In this review, studies reported a low incidence (0%-8%) of adverse effects in participants utilizing virtual reality therapy; however, it should be reiterated that only 6 studies screened for side effects.

Some of the main side-effects associated with virtual reality were nausea, vomiting, eye strain, and dizziness; cumulatively referred to as cybersickness [60]. The most widely accepted theory explaining cybersickness relates to the Sensory Conflict Theory. This refers to the discrepancy which occurs between the ocular and vestibular systems when the senses do not receive the usual sensory feedback that would be expected in such a scenario. This lack of synchronization is believed to cause cybersickness [61,62].

This is of relevance to the clinician using the technology for several reasons. For one, this alludes to a vulnerable population of patients who are susceptible to these side effects and who should be excluded from its use, such as patients with vestibular abnormalities, with seizure disorders, and who experience migraines or headaches [63]. Additionally, this alludes to a number of methods that can be considered to reduce the incidence of these effects during use of the technology. Although a discussion of these is beyond the scope of this review, the following articles provide adequate reference material [64-68].

### Virtual Reality Technological Perspectives

Most virtual reality interactive hardware consists of a combination of a head mounted display, built-in biaural headphones for sound, and a trackpad or joystick for manipulation or navigation of the virtual environment, to provide the user with an immersive experience [69].

### Head Mounted Display and User Control

In our review, all studies utilized a head mounted display for the administration of virtual reality to participants. These ranged from portable hardware, such as a helmet or piece of cardboard, to more sophisticated hardware systems, where participants were connected to an external processing unit.

A head mounted display displays content via 2 screens placed in front of the user’s eyes which are stereoscopic in nature. The images displayed are angled to provide a variation in depth perception, which is interpreted by the brain as having 3D characteristics and features. In addition, the head mounted displays track user interaction in real time, which updates the virtual content that is reflected to the user simultaneously [70]. This can be either through tracking head orientation or position of the user’s physical movements, as well as walking and jumping [69,71]. None of the studies in this review used systems capable of positional tracking. This is understandable, considering that clinical procedures, including those examined, typically necessitate controlled patient positioning.

### Virtual Reality Content

In this study, 50% of the content was an active form of virtual reality, which entailed an element of interaction with the environment by the participant. In contrast, the remainder administered a passive form of virtual reality, where participants could only observe the content. This is worth mentioning as the available evidence suggests that the analgesia afforded by active virtual reality is significantly more than that offered by the passive form [72-74]. No study in our review, however, explicitly investigated this difference.

### Limitations

The findings of this review should be interpreted considering the following limitations.

First, it is important to note that the results of this review could be influenced by publication bias. Particularly when considering that Fanelli et al [75] demonstrated that approximately 90% of literature in the fields of psychiatry, psychology, and clinical medicine report positive findings. As a result, the performance of virtual reality as an analgesic could be overestimated. Second, this limitation is further exacerbated by the use of a narrative approach employing descriptive statistics, as was the case for this review. Similarly, the search strategy was restricted to health databases given the interest in clinically relevant findings. However, in doing so, we neglected technical databases such as IEEE and ACM, which might have had further data of relevance. Unfortunately, this is an issue that plagues research in the field of medical technology as it attempts to incorporate the two very separate domains of health and technology. To bridge this, we have provided information in the discussion to supplement areas where more technical knowledge may have been required. Third, as the sample sizes of the studies included in this review were generally small and based on very specific inpatient populations, the generalizability of the findings may be limited. In addition to this, a large variety of measurement tools were implemented to quantify the outcome of pain. This unfortunately precludes meta-analysis of the data, which would have otherwise been useful to quantify accurate treatment effects.

### Areas for Future Research

The systematic review highlighted the need for further large-scale prospective studies to be conducted in order to investigate the efficacy of virtual reality therapy in facilitating analgesia and anxiolysis. Additionally, this review also highlighted the need for investigators to screen patients for cybersickness-related side effects as part of their study design. Finally, it is also suggested that future clinical studies explore the differences between active and passive forms of virtual reality in facilitating analgesia.

### Conclusion

This review sheds light on the efficacy of utilizing virtual reality therapy for the reduction of acute pain and procedural anxiety within an inpatient setting to hopefully offer a novel and practical perspective on the matter. Furthermore, it demonstrated a low incidence of adverse side effects in the populations being sampled. For clinical use, there appeared to be a preference for head mounted display to display virtual reality content. Although no differences between active and passive virtual reality were identified in this review, the literature appeared to suggest that active virtual reality would facilitate a higher level of analgesia in comparison to that facilitated by passive virtual reality [72-74]. Finally, although there was no evidence found to suggest an effect of virtual reality therapy on physiological parameters (autonomic arousal) as a surrogate biomarker of pain, this review also suggested merit in continuing this line of investigation in a rigorous and reproducible manner. It is hoped that this study serves to inform future trials to assess the efficacy of virtual reality in the treatment of acute pain.

## Appendix

Multimedia Appendix 1 General description of studies included in the review.

Multimedia Appendix 2 Technical specification of devices.

Multimedia Appendix 3 Critical appraisal of studies which failed to demonstrate a significant reduction in pain scores.
