# Supplementary material for: The Effectiveness of Virtual Reality in Managing Acute Pain and Anxiety for Medical Inpatients: Systematic Review
Source: J Med Internet Res. 2020 Nov 2;22(11):e17980. doi: 10.2196/17980 (PMC7669439; doi:10.2196/17980)
Supplement: Multimedia Appendix 1 [file jmir_v22i11e17980_app1.docx]

**Table S1**: General description of studies included in the review.

| **Author** | **Year** | **Study Design** | **Population** | **Setting** | **Number of Patients** | **Age Range** | **Procedure** | **Country** | **Study Quality** |
| --- | --- | --- | --- | --- | --- | --- | --- | --- | --- |
| **Chad et al.^35^** | 2018 | Cohort Study | Children | Hospital | 17 | N.A. | Routine Immunization | USA | *Modified Black’s and Downs score: 9*  Main outcomes of the study were not clearly described in the introduction or methods sections. |
| **Chan et al.^24^** | 2007 | Crossover Trial | Children | Hospital | 8 | 4 - 8 | Burns Dressing Change | Hong Kong | *Modified Black’s and Downs score: 6*  Study did not:   - Provide estimates of random variability; - Report actual probability values; and - Use participants representative of the recruitment population (n=8; 7 male and 1 female). |
| **Chau et al.^34^** | 2018 | Retrospective Chart Review | Children | Hospital | 14 | 5 - 13 | Botulinum Injections for Spasticity | USA | *Modified Black’s and Downs score: 8*  Study did not provide estimates of random variability nor did it report actual probability values. |
| **Frey et al.^37^** | 2018 | Crossover Trial | Adults | Hospital | 28 | 18 - 45 | Unmedicated Labour | USA | *Modified Black’s and Downs score: 9*  Study did not report probability values (p-value) for its main outcomes, instead providing mean data only. |
| **Gerceker et al.^33^** | 2017 | RCT | Children | Hospital | 121 | 7 - 12 | Venepuncture | Turkey | *Modified Black’s and Downs score: 10*  Study fulfilled all requirements as per the modified checklist. |
| **Gershon et al.^23^** | 2004 | RCT | Children Adolescents | Hospital | 59 | 7 - 19 | Port Access | USA | *Modified Black’s and Downs score: 8*  Study did not provide estimates of random variability nor did it report actual probability values, eg. p=0.035 instead of p<0.05. |
| **Glennon et al.^36^** | 2018 | Interventional Study | Adults | Hospital | 97 | >18 | Bone Marrow Aspiration & Biopsy | USA | *Modified Black’s and Downs score: 9*  Study did not report actual probability values, eg. p=0.035 instead of p<0.05. |
| **Gold et al.^28^** | 2017 | RCT | Children | Hospital | 143 | 10 - 21 | Venepuncture | USA | *Modified Black’s and Downs score: 9*  Study did not provide estimates of random variability for its main outcomes. |
| **Hoffman et al.^17^** | 2008 | Case Control | Children Adolescents Adults | Hospital | 11 | 9 - 40 | Hydrotherapy Wound Debridement | USA | *Modified Black’s and Downs score: 8*  Study did not provide estimates of random variability for its main outcomes, nor were the participants who were prepared to participate representative of the recruitment population. |
| **McSherry et al.^29^** | 2017 | RCT | Adults | Hospital | 18 | 22 - 53 | Wound Care Procedures | USA | *Modified Black’s and Downs score: 7*  Study did not provide estimates of random variability for its main outcomes, nor were the participants who were prepared to participate representative of the recruitment population (67% were substance abusers). |
| **Mosso-Vasquez et al.^16^** | 2014 | Cohort Study | Adults | Hospital | 67 | N.A. | Post Cardiac Surgery | Mexico | *Modified Black’s and Downs score: 8*  Study did not provide estimates of random variability nor did it report actual probability values, eg. p=0.035 instead of p<0.05. |
| **Mosso-Vasquez et al.^32^** | 2018 | RCT | Adults | Hospital | 44 | >18 | Resection of Lipoma | Mexico | *Modified Black’s and Downs score: 7*  Study did not:   - Describe the main outcomes in the introduction or methods sections; - Describe the characteristics of the patients included; and - Provide estimates of random variability (used Z-test) |
| **Nilsson et al.^18^** | 2009 | RCT | Children Adolescents | Hospital | 48 | 5 - 18 | Venepuncture/Port Access | Sweden | *Modified Black’s and Downs score: 9*  Main outcomes of the study were not clearly described in the introduction or methods sections. Also, random variability estimates were only applied to HR. |
| **Piskorz et al.^30^** | 2017 | Case Control | Children Adolescents | Hospital | 38 | 8 - 14 | Venepuncture | Poland | *Modified Black’s and Downs score: 8*  Study did not provide estimates of random variability nor did it report actual probability values, eg. p=0.035 instead of p<0.05. |
| **Shoorab et al.^26^** | 2015 | RCT | Adults | Hospital | 32 | 18 - 34 | Episiotomy Repair | Iran | *Modified Black’s and Downs score: 9*  Study did not provide estimates of random variability for its main outcomes. |
| **Tashjian et al.^31^** | 2017 | Cohort Study | Adults | Hospital | 100 | 32 - 72 | Acute pain for various pathologies | USA | *Modified Black’s and Downs score: 9*  Main outcomes of the study were not clearly described in the introduction or methods sections. |
| **Walker et al.^25^** | 2014 | RCT | Adults | Hospital | 45 | 18 - 70 | Flexible Cystoscopy | USA | *Modified Black’s and Downs score: 8*  Study did not provide estimates of random variability nor did it report actual probability values, eg. p=0.035 instead of p<0.05. |
| **Yun Hua et al.^27^** | 2015 | RCT | Children | Hospital | 65 | 4 - 16 | Chronic Lower Limb Wound Dressing Change | China | *Modified Black’s and Downs score: 9*  Study did not provide estimates of random variability for its main outcomes. |

RCT- Randomised controlled trial
