# Supplementary material for: The Effectiveness of Virtual Reality in Managing Acute Pain and Anxiety for Medical Inpatients: Systematic Review
Source: J Med Internet Res. 2020 Nov 2;22(11):e17980. doi: 10.2196/17980 (PMC7669439; doi:10.2196/17980)
Supplement: Multimedia Appendix 2 [file jmir_v22i11e17980_app2.docx]

**Table S2:** Technical specification of devices utilised in study.

| **Author** | **VR Content Type** | **VR Content** | **Goggles/HMD** | **Display** | **Weight** | **Field of View** | **Computer** | **Video Card** | **Controller** |
| --- | --- | --- | --- | --- | --- | --- | --- | --- | --- |
| **Chad et al.^35^** | Environment | Roller coaster ride, helicopter ride or hot-air balloon ride | Tepoinn 3D VR Headset | Smartphone Display | 13.8 oz | 100–120 degrees | Unspecified | Unspecified | Unspecified |
| **Chan et al.^24^** | Game | Ice-cream World | I-glasses | Twin LCD displays with 640 x 480 resolution | 2.4 oz | 35 degrees | Unspecified | Unspecified | Unspecified |
| **Chau et al.^34^** | Movie | 360° videos sourced from YouTube | Generic unit such as the Google Cardboard | Smartphone Display | Unspecified | Unspecified | Unspecified | Unspecified | Unspecified |
| **Frey et al.^37^** | Environment | Ocean Rift | Samsung Gear Oculus with Samsung Galaxy S7 phone | 1440 x 1280 per eye | 12.1 oz | 101 degrees | N.A | N.A | Oculus Controller |
| **Gerceker et al.^33^** | Movie | Magic English Disney Family, Princess Sofia’s Secret Library & Dinosaur Animation Cartoon for Children | Samsung Gear Oculus with Samsung Galaxy S5 Note phone | 1440 x 1280 per eye | 12.1 oz | 101 degrees | N.A | N.A | N.A |
| **Gershon et al.^23^** | Game | Gorilla World | Unspecified VR HMD | Unspecified | Unspecified | Unspecified | Unspecified | Unspecified | Computer Joystick |
| **Glennon et al.^36^** | Environment | Relaxing Nature Scenes | ezVision X4 VR Goggles | 640 x 480 per eye | 2.5 oz | 32 degrees | N.A. | N.A. | N.A. |
| **Gold et al.^28^** | Game | Bear Blast | Samsung Galaxy S6 and Samsung Gear VR or Google Pixel and Merge VR goggles | 1440 x 1280 per eye | 12.1 oz | 101 degrees | N. A | N. A | Trackpad |
| **Hoffman et al.^17^** | Game | Snow world | Custom VR Helmet with InFocus LP70 Projectors | Unspecified | Unspecified | Unspecified | Dell 530 Workstation with 2GHz CPUs, 2GB RAM, Toroid Isolation Transformer | GeForce 6800 | Microsoft Sidewinder Joystick |
| **McSherry et al.^29^** | Game | Snow world | NVISINC MX 90 Goggles | Unspecified | Unspecified | Unspecified | Lenovo T510 Thinkpad, Intel Core i7 | Intel HD Graphics | Computer Mouse |
| **Mosso-Vasquez et al.^16^** | Environment | Cliff, Dream Castle, Enchanted Forest, Icy Cool World and Drive, Walk, Bike | Unspecified VR HMD | Unspecified | Unspecified | Unspecified | Unspecified | Unspecified | N. A |
| **Mosso-Vasquez et al.^32^** | Environment | Enchanted Forest and Magic Cliff | Illusion Mask 3D Virtual Reality Glasses & Apple iPhone 6 | Unspecified | Unspecified | Unspecified | Unspecified | Unspecified | Unspecified |
| **Nilsson et al.^18^** | Game | Hunt of the Diamonds | X3D-20 | 1600 x 1200 resolution | Unspecified | Unspecified | Unspecified | Unspecified High-End graphics card | GyroRemote remote control |
| **Piskorz et al.^30^** | Game | Unspecified | Oculus Rift DK2 HMD | Twin OLED displays with 960 x 1080 resolution | 15.5 oz | 100 degrees | Unspecified | Unspecified | Oculus Controller |
| **Shoorab et al.^26^** | Movie | Dolphins & Whales | Vuzix Wrap 920 | Twin LCD Displays at 640x480 resolution | 3 oz | 31 degrees | N. A | N. A | External remote-control device |
| **Tashjian et al.^31^** | Environment | Pain RelieVR | Samsung Gear Oculus with Samsung Galaxy S7 phone | 1440 x 1280 per eye | 12.1 oz | 101 degrees | N. A | N. A | Trackpad |
| **Walker et al.^25^** | Game | Snow world | Unspecified VR Helmet | Unspecified | Unspecified | Unspecified | Unspecified | Unspecified | Trackball Hand Controller |
| **Yun Hua et al.^27^** | Game | Ice Age 2 | eMagin Z800 3DVISOR HMD | Twin OLED displays at 800 x 600 resolution | 8 oz | 40 degrees | Lenovo-Y430 Laptop, 2.5Hz Intel Core i7, 8GB DDR3 | NVIDIA GTX 850M | BETOP TE BTP-2185 Joystick |
