# Supplementary material for: The Effectiveness of Virtual Reality in Managing Acute Pain and Anxiety for Medical Inpatients: Systematic Review
Source: J Med Internet Res. 2020 Nov 2;22(11):e17980. doi: 10.2196/17980 (PMC7669439; doi:10.2196/17980)
Supplement: Multimedia Appendix 3 [file jmir_v22i11e17980_app3.docx]

**Appendix C:** Critical appraisal of studies which failed to demonstrate a significant reduction in pain scores.

In construing the findings of *Nilsson et al.* in children undergoing venepuncture or port access, a number of factors need to be taken into consideration. Firstly, the study was underpowered to detect a treatment effect. In addition, the children in this study were well familiarized with the procedure and may have developed coping mechanisms which could account for the insignificant reduction in pain and observed distress [1].

*Walker et al.* observed no significant difference in pain reduction in the VR group compared to the control group in their study which was conducted on men undergoing flexible cystoscopy. All patients however, had a local anaesthetic applied (2% lidocaine jelly lubricant) during the procedure as well as verbal reassurances from the conducting urologist which may have influenced their reporting of pain [2]. This could have introduced a measurement bias into their study,

*McSherry et al.* found no significant differences in pain scores before and after the procedure in adults undergoing wound care procedures. There was, however, a high incidence of substance abuse history (67%) amongst their participants, which the authors theorized may have altered their pain perception and subsequent reporting of pain [3].

*Glennon et al*. found no significant differences in pain and anxiety between the VR group and the control group during bone marrow aspiration and biopsy. The authors postulated that since their recruitment incorporated patients who had experienced multiple bone marrow aspiration and biopsy procedures that this could have affected their reporting and perception pain [4].

References

1. Nilsson S, Finnstrom B, Kokinsky E, Enskar K. The use of Virtual Reality for needle-related procedural pain and distress in children and adolescents in a paediatric oncology unit. *European journal of oncology nursing : the official journal of European Oncology Nursing Society.* 2009;13(2):102-109. doi:10.1016/j.ejon.2009.01.003

2. Walker MR, Kallingal GJ, Musser JE, Folen R, Stetz MC, Clark JY. Treatment efficacy of virtual reality distraction in the reduction of pain and anxiety during cystoscopy. *Military medicine.* 2014;179(8):891-896. doi:10.7205/milmed-d-13-00343

3. McSherry T, Atterbury M, Gartner S, Helmold E, Searles DM, Schulman C. Randomized, Crossover Study of Immersive Virtual Reality to Decrease Opioid Use During Painful Wound Care Procedures in Adults. *Journal of burn care & research : official publication of the American Burn Association.* 2017. doi:10.1097/bcr.0000000000000589

4. Glennon C, McElroy SF, Connelly LM, et al. Use of Virtual Reality to Distract From Pain and Anxiety. *Oncology nursing forum.* 2018;45(4):545-552. doi:10.1188/18.Onf.545-552
